# Exploring the Synergy between Nano-Formulated Linezolid and Polymyxin B as a Gram-Negative Effective Antibiotic Delivery System Based on Mesoporous Silica Nanoparticles

**DOI:** 10.3390/nano14020228

**Published:** 2024-01-20

**Authors:** Ismael Otri, Serena Medaglia, Ramón Martínez-Máñez, Elena Aznar, Félix Sancenón

**Affiliations:** 1 Instituto Interuniversitario de Investigación de Reconocimiento Molecular y Desarrollo Tecnológico (IDM), Universitat Politècnica de València, Universitat de València, Camino de Vera s/n, 46022 Valencia, Spain; ismaelotri84@gmail.com (I.O.); sermed@idm.upv.es (S.M.); rmaez@qim.upv.es (R.M.-M.); 2 CIBER de Bioingeniería, Biomateriales y Nanomedicina (CIBER-BBN), Instituto de Salud Carlos III, 46022 Valencia, Spain; 3 Unidad Mixta de Investigación en Nanomedicina y Sensores, Instituto de Investigación Sanitaria La Fe, Universitat Politècnica de València, 46026 Valencia, Spain; 4 Unidad Mixta UPV-CIPF de Investigación en Mecanismos de Enfermedades y Nanomedicina, Centro de Investigación Príncipe Felipe, Universitat Politècnica de València, 46012 Valencia, Spain; 5 Departamento de Química, Universitat Politècnica de València, Camino de Vera s/n, 46022 Valencia, Spain

**Keywords:** gated materials, antimicrobials, polymyxin B, linezolid

## Abstract

Antimicrobial resistance is a current silent pandemic that needs new types of antimicrobial agents different from the classic antibiotics that are known to lose efficiency over time. Encapsulation of antibiotics inside nano-delivery systems could be a promising, effective strategy that is able to delay the capability of pathogens to develop resistance mechanisms against antimicrobials. These systems can be adapted to deliver already discovered antibiotics to specific infection sites in a more successful way. Herein, mesoporous silica nanomaterials are used for an efficient delivery of a linezolid gram-positive antibiotic that acts synergistically with gram-negative antimicrobial polymyxin B. For this purpose, linezolid is encapsulated in the pores of the mesoporous silica, whose outer surface is coated with a polymyxin B membrane disruptor. The nanomaterial showed a good controlled-release performance in the presence of lipopolysaccharide, found in bacteria cell membranes, and the complete bacteria *E. coli DH5α.* The performed studies demonstrate that when the novel formulation is near bacteria, polymyxin B interacts with the cell membrane, thereby promoting its permeation. After this step, linezolid can easily penetrate the bacteria and act with efficacy to kill the microorganism. The nano-delivery system presents a highly increased antimicrobial efficacy against gram-negative bacteria, where the use of free linezolid is not effective, with a fractional inhibitory concentration index of 0.0063 for *E. coli*. Moreover, enhanced toxicity against gram-positive bacteria was confirmed thanks to the combination of both antibiotics in the same nanoparticles. Although this new nanomaterial should be further studied to reach clinical practice, the obtained results pave the way to the development of new nanoformulations which could help in the fight against bacterial infections.

## 1. Introduction

The World Health Organization (WHO) considers the raising resistance of microorganisms to antibiotics as a coming pandemic [1]. Over the years, microorganisms have been becoming more resistant to many existing antimicrobials. It is foreseen that everyday infections will not be able to be treated through current antibiotics in the near future. Antimicrobial resistance (AMR) causes at least 700,000 deaths around the world each year. These numbers are estimated to reach 10 million by 2050 [2,3]. Over the years, pathogens have been able to increase their resistance to antibiotics through mutation and selection concepts [4,5,6]. This has been accelerated through the inappropriate use of these medicines as well as the lack of research and money invested by governments and the pharmaceutical industry into the development of new antibiotics. Over the last 10 years, new molecular-based antibiotics have not been discovered as it is a slow and expensive process that takes 10–15 years and over EUR 1 billion [7]. Currently, most available antibiotics are losing their efficiency. For example, according to a WHO report in November 2021, the *Staphylococcus aureus* bacterium, which is part of our skin flora and a common cause of infections both in the community and in healthcare facilities, is becoming resistant to most antibiotic treatments. For example, people suffering from infections caused by methicillin-resistant *S. aureus* (*MRSA*) are 64% more likely to die. Huge efforts are needed to tackle AMR to avoid the collapse of the world health systems, the economic impact, and the number of deaths caused by infections. In addition to government investment and changing human behaviour by raising awareness in people about the problem and the current status of the situation, research and development of new antimicrobial medicines, vaccines, and diagnostic tools are urgently required, especially for gram-negative bacteria such as *Pseudomonas aeruginosa*, carbapenem-resistant *Enterobacteriaceae*, or *Acinetobacter baumannii* for which most current antibiotics are far from being sufficiently efficient.

Currently, there are several strategies for the development of new antimicrobials thanks to the discovery of new biological, biochemical, and chemical tools such as bacteriocins, phage therapy, antimicrobial heavy metals, lysins, lactam antibiotics [8,9], and antimicrobial peptides [10]. Also within the scope of this research area, the encapsulation of antibiotics inside porous nanomaterials has emerged as a promising solution to slow down the rapid increase in resistance [11,12]. Antibiotic confinement in porous materials was found to decrease the stimulation and activation of the resistance mechanism of a resistance organism [13]. At the same time, this strategy decreases the side effects and toxicity caused by some antibiotics [14,15].

For example, gram-negative bacteria develop resistance to antibiotics faster than gram-positive ones due to their membrane structure being composed of a thin layer of inner peptidoglycan and an outer negatively charged lipopolysaccharide (LPS) membrane, which provides high protection against a wide range of antibiotics [16]. Most of the antibiotics used to treat gram-negative infections cause toxicity by disturbing the LPS layer through interaction or degradation. As mentioned above, using nanomaterials for the delivery of antibiotics could be a promising strategy for protecting antimicrobials from resistance. Nanomaterials can provide protection against enzyme degradation in cells and increase their circulation time in body fluids while the antibiotics are encapsulated inside the nanomaterials. The nanoparticle surface also presents an opportunity to improve antimicrobial efficiency. Through the functionalization of the outer surface by different molecules, such as targeting agents for specific delivery to infective cells, a decrease in the side effects can be achieved. Moreover, coating the outer surface with hydrophilic and penetrating molecules can improve the ability to cross body membranes and to extend circulation lifetime [17]. Nanomaterials open the possibility to use antibiotics that are hydrophobic, present very short circulation time or need a high dose to be effective. Several nanomaterials were used for the delivery of antibiotics including polymers [18], liposomes [19], hydrogels [20], nanoemulsions [21], lipid nanoparticles [22] and others [23]. Mesoporous silica nanoparticles have been investigated over the last decade for delivery applications due to their excellent properties such as a high surface area combined with high cargo capability, their ease of synthesis and functionalization with a wide range of molecules using simple chemistry. Moreover, the inertness, thermal stability, and homogeneity of the inner porous system are features which make these nanomaterials excellent candidates [24] for antimicrobial delivery [25]. In most cases, antibiotics are first loaded into the inner pores of mesoporous silica, and then the external surface is coated with different molecules and targeting agents. Coating the outer surface with bulk species has been found to be effective for inhibiting the release of the loaded antibiotics. At the same time, these blocking molecules can work as stimuli-responsive gates which can trigger cargo delivery. In such systems, antibiotic cargo release is induced in the presence of target stimuli and in the infection site [26]. In this context, many gated mesoporous silica nanomaterials have been reported to respond to physical stimuli, such as temperature and electric or magnetic fields, or to a change in chemical conditions, such as the pH, redox environment, or the presence of specific molecules or enzymes [27,28]. Bearing in mind our previous experience in the preparation of gated nanomaterials as hybrid systems for sensing and delivery applications [29,30], herein we develop new mesoporous silica nanoparticles for the efficient delivery of the linezolid antibiotic to bacteria. Linezolid belongs to the oxazolidinone family and it is an aggressive antibiotic effective against serious infections mainly caused by gram-positive bacteria in the skin and soft tissues [31]. At a physiological pH, linezolid has moderate solubility that is around 3.0 mg/mL [32]. Due to this limited efficacy, solubility, and side effects, linezolid presents certain limitations that could be addressed by using a smart delivery system. To achieve our objective, we also focused on the FDA-approved antibiotic polymyxin B (PMB) [33]. Polymyxin B is a bactericidal drug which acts on the outer membrane of bacteria by destabilizing the phospholipids and lipopolysaccharides (LPS) present. Thanks to its cationic and branched structure, it has excellent features that can be used to cap anionic surfaces for gating purposes. This work aims to develop, for the first time, a novel, repurposed linezolid-PMB nanoformulation to broaden the application of linezolid with more efficacy in gram-negative bacteria.

**Scheme 1:**
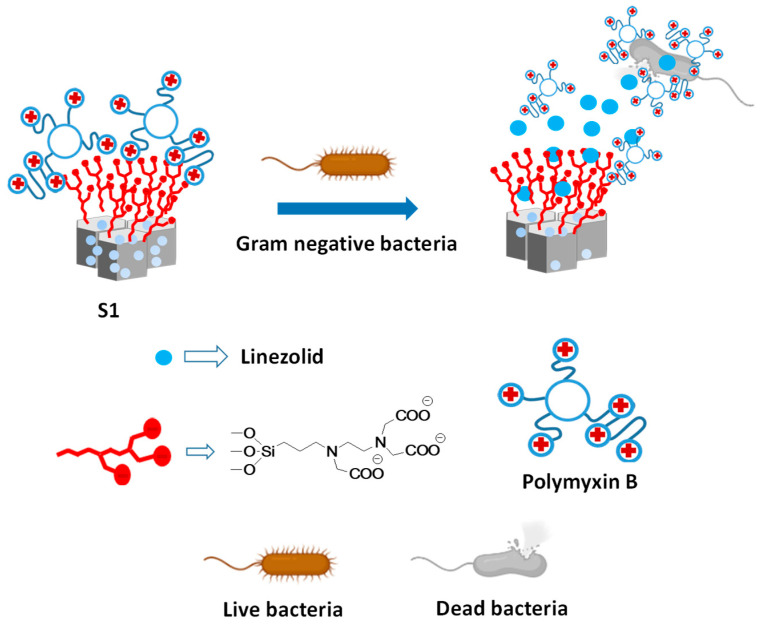
Schematic representation of the prepared nanomaterial **S1** and its release mechanism.

The silica nanomaterial used for the effective delivery of linezolid consists of ordered mesoporous nanoparticles loaded with the antibiotic linezolid and functionalized with carboxylate moieties able to interact with the positively charged antimicrobial agent polymyxin B. When PMB interacts with carboxylates, it is able to cap the pores and act as a stimuli-responsive molecular gate. Thanks to PMB’s ability to interact in a highly affinitive way with LPS (which can be found on the bacteria’s outer surface [34,35,36]), PMB is expected to be displaced from the nanomaterial’s surface in the presence of bacteria, triggering the on-site release of linezolid. It was expected that this synergic action facilitates the killing of bacteria, as depicted in Scheme 1.

## 2. Materials and Methods

### 2.1. Chemicals

Tetraethylorthosilicate (TEOS), n-cetyltrimethyl ammonium bromide (CTABr), sodium hydroxide (NaOH), rhodamine B, tris(hydroxymethyl), aminomethane (TRIS), endotoxin-free Dulbecco’s PBS (1X), and Lipopolysaccharides from *Escherichia coli* O55:B5 (LPS) were purchased from Sigma-Aldrich Química (Madrid, Spain). LPS from R. sphaeroides (LPS-RS) was purchased from InvivioGen, (San Diego, CA, USA). N-[(3-trimethoxysilyl) propyl] ethylene diamine triacetic acid trisodium salt was purchased from Fluorochem, (Hadfield, UK). Polymyxin B sulfate (PMB) was purchased from Tokyo Chemical Industry Co., Ltd. (TCI) (Tokyo, Japan). Linezolid was purchased from Santa Cruz Biotechnology, Inc. (Dallas, TX, USA). Analytical grade solvents were purchased from Scharlab (Barcelona, Spain). All products were used as received.

### 2.2. General Techniques

Transmission electron microscopy (TEM), N_2_ adsorption–desorption isotherms, dynamic light scattering (DLS), and powder X-ray diffraction (PXRD) were used to characterize the prepared materials. A JEOL JEM-1010 microscope (JEOL, Tokyo, Japan) was used for TEM image acquisition. For sample visualization, a suspension of 1 mg mL^−1^ in distilled water was prepared and placed on carbon film-supported copper electron microscopy grids. Samples were left drying for at least 24 h. PXRD measurements were taken using a Bruker D8 Advance diffractometer (Bruker, Billerica, MA, USA) (Cu Kα radiation). Thermogravimetry of the materials was performed using a TGA/SDTA 851e balance from Mettler Toledo (Mettler Toledo Inc., Schwarzenbach, Switzerland). Loss weight in an oxidant atmosphere (air, 80 mLmin^−1^) was registered within a dynamic step in which an increase of 10 °C min^−1^ was applied in an interval from 20 °C to 1000 °C. Then, temperature was maintained at 1000 °C for an extra 5 min. Porosimetry studies were performed using nitrogen and Tristar II Plus equipment from Micromeritics (Micromeritics Instrument Corporation, Norcross, GA, USA). Sample degasification was performed overnight at 90 or 120 °C. The specific surface areas were calculated from the adsorption data within the low-pressure range using the BET (Brunauer–Emmett–Teller) model. Pore size was determined following the BJH (Barrett–Joyner–Halenda) method. DLS experiments were performed using a Malvern ZetaSizer Nano ZS (Malvern Panalytical, Malvern, UK).

### 2.3. Synthesis of Mesoporous Silica Nanoparticles (MSNs)

N-cetyltrimethylammonium bromide (CTABr, 1.00 g, 2.74 mmol) was first dissolved in 480 mL of deionized water. Then, 3.5 mL of NaOH 2.00 M in deionized water was added to the CTABr solution, followed by adjusting the solution temperature to 80 °C. TEOS (5 mL, 25.7 mmol) was then added dropwise to the surfactant solution. The mixture was allowed to stir for 2 h to give a white suspension. Finally, the solid was centrifuged, washed with deionized water, and dried at 60 °C (MSNs as-synthesized). To prepare the final mesoporous material, the as-synthesized solid was calcined at 550 °C in an oxygen atmosphere for 5 h in order to remove the template phase.

### 2.4. Synthesis of ***S1***

A total of 750 mg of template-free MCM-41 was suspended in 10 mL of an anhydrous acetonitrile of 375 mg of Linezolid in a round-bottomed flask, (1.1 mmol of Linezolid/g MCM-41). After 24 h stirring at room temperature, 15 mmol/g MCM-41 of N-[(3-trimethoxysilyl)propyl]ethylenediamine triacetic acid trisodium salt was added and the mixture was stirred for 5.5 h at room temperature. Then, PMB (2.3 mmol/g) was added to the suspension drop by drop and allowed to stir overnight. Finally, this solid was filtered and washed with PBS plus 5% ACN to remove the unreacted alkoxysilane and the linezolid remaining outside the pores. The final solid, which is called **S1,** was dried under vacuum at ambient temperature for 12 h.

### 2.5. Synthesis of ***S1-Rh***

In a typical synthesis, 750 mg of template-free MCM-41 was suspended in a solution of 340 mg of Rhodamine B dye in 10 mL of Mili Q water in a round-bottomed flask (0.8 mmol of dye/g MCM-41). After 24 h of stirring at room temperature, 15 mmol/g MCM-41 of N-[(3-trimethoxysilyl)propyl]ethylenediamine triacetic acid trisodium salt was added and the mixture was stirred for 5.5 h at room temperature. Then, PMB (2.3 mmol/g) was added to the suspension and allowed to stir for 2 h. This suspension was stirred for 1 h at room temperature. Finally, this solid was filtered and washed with PBS in order to remove the unreacted alkoxysilane and the dye remaining outside the pores. The final solid **S1-Rh** was dried under vacuum at ambient temperature for 12 h.

### 2.6. Release Test with Bacterial and Free LPS

In vitro dye-release studies were carried out with solid **S1-Rh** to test the correct performance with bacteria (*Escherichia coli*, DH5α strain). In a typical experiment, 3000 µg of solid **S1-Rh** was suspended in PBS at pH 7.2 and divided into three aliquots. 10^5^ cell ml^−1^ of *E. coli*, 2500 µg/mL of LPS and blank were added, respectively. All suspensions were incubated at room temperature, and at given times fractions of suspensions were taken and filtered using a 0.22 µm filter to remove the solid. The delivery of the rhodamine B dye was then monitored through the fluorescence emission band at 610 nm (λ_exc_ = 453 nm).

### 2.7. E. coli DH5α Culture Conditions

For viability studies, bacteria (*E. coli*) cell culture DH5α was used as well as general enrichment media type LB broth and LB agar from Laboratories Conda. All reagents were used following the manufacturer’s conditions.

DH5α cells were cultured in an LB medium at 37 °C overnight with continuous stirring, then 1 mL of culture were collected through centrifugation for 30 s at 13,000 rpm and resuspended in 1 mL of PBS. Then, a dilution of 10^−5^ cells/mL was prepared depending on OD_620_.

### 2.8. Cell Viability Studies with E. coli DH5α (Clonogenic Viability Assays to Determine ***S1***, Free PMB, and Linezolid Cytotoxicity)

Serial dilutions for **S1**, free PMB, free Linezolid, and mixed PMB and Linezolid (from 1000 to 10^−7^ µg/mL) were prepared in PBS (pH 7.2) and then incubated with 10^−5^ cells per mL^−1^ of *E. coli* DH5α with shaking of 150 rpm at 37 °C for 10 min. Then, an aliquot of 100 µL, diluted at a ratio of 1:10, was used to obtain countable CFUs on the plates, and then 100 µL of each of these was seeded into the LB agar plate from each dilution (each concentration duplicated). After that, all the plates were incubated at 37 °C overnight. Then, colony formation units (CFUs) were counted in each plate, and the percentage of cytotoxicity was determined in comparison with the negative control as 100% viability. The concentration of free linezolid/PMB was 1 mg/mL of each of these mixed together.

### 2.9. Minimum Inhibitory Concentrations (MICs)

MIC were determined through the standard broth microdilution method in a 96-well plate format. A total of 10^−5^ cells per mL^−1^ of bacteria were prepared in LB broth for *S. aureus* and *E.coli,* and then 100 µL was filled in 96-well plates for each of the bacteria separately. Serial dilutions (1000 µg to 10^−7^ mL^−1^) of free linezolid, PMB and **S1** were prepared and aliquoted 200 µL in each well then added to 10^−5^ cells of bacteria per mL^−1^. Each 96 well plate was prepared for the type of bacteria to prevent cross-contamination. Then, the negative control (media with bacteria only) and blank (media with antibacterial agent only) were performed as well; all plates were incubated over night at 37 °C and repeated three times. Bacterial growth was determined turbidimetrically (OD_620_) using an Elisa plate reader by calculating the mean of viability for each concentration.

### 2.10. Fractional Inhibitory Concentration Index (FICI)

To study the synergic effect of the **S1** nanomaterial, the fractional inhibitory concentration index (FICI) was calculated according to the following formula:

$${FIC}_{Lin}=\frac{S1}{{MIC}_{Lin}}$$

$${FIC}_{PMB}=\frac{S1}{{MIC}_{PMB}}$$

$$FICI={FIC}_{Lin}+{FIC}_{PMB}$$

where **S1** is the MIC value for the solid **S1** calculated through the standard microdilution method, and MIC_Lin_ and MIC_PMB_ are the minimum inhibitory concentrations of linezolid and PMB, respectively.

A FICI ≤ 0.5 value indicates a synergistic effect of the combination, 0.5 < FICI ≤ 1 can be considered as an additive effect, 1 < FICI > 4 represents an indifferent effect, and FICI > 4 can be considered as antagonistic behaviour.

## 3. Results and Discussion

### 3.1. Synthesis and Characterization of Materials

To obtain the **S1** nanomaterial, ordered mesoporous nanoparticles were prepared through known procedures using n-cetyltrimethylammonium bromide (CTABr) as a template and tetraethylorthosilicate (TEOS) as a silicon precursor [37]. After removing the surfactant through calcination, the empty pores were loaded with linezolid and the external surface was functionalized using n-[(3-trimethoxysilyl)propyl]ethylene diamine triacetic acid trisodium salt to obtain a negatively charged surface. The final nanomaterial was obtained by adding PMB. The electrostatic interaction between the anionic charge of carboxylate moieties and the cationic PMB block the pores of the nanoparticles, as shown in Scheme 1 (see Materials and Methods section for the detailed procedures).

**Figure 1:**
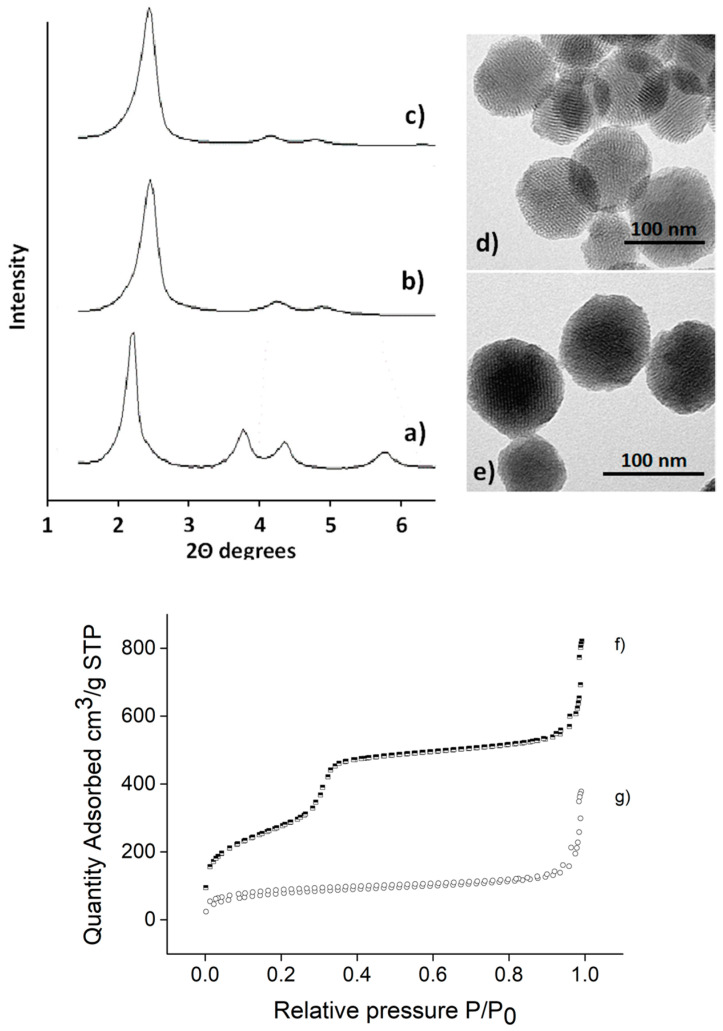
(**Left**) powder X-ray patterns of (**a**) as-synthesized MSN, (**b**) calcined MSN, and (**c**) solid **S1**. (**Right**) representative TEM images of (**d**) the calcined MSN sample and (**e**) solid **S1**. N_2_ adsorption–desorption isotherms are shown for (**f**) calcined MSN and (**g**) solid **S1**.

Different characterization methods were used to examine the correct preparation of the nanomaterial. First, the starting mesoporous silica nanoparticles (MSNs), before and after surfactant removal through calcination at 550 °C, were characterized using powder X-ray diffraction (PXRD). Transmission electron microscopy (TEM) was used to study the size of nanoparticles, and isotherm N_2_ adsorption–desorption experiments were performed to assess the total surface area and pore size of the MSNs. As can be appreciated in Figure 1a, PXRD studies of the as-made nanomaterial showed the four typical low-angle reflections [38] of a hexagonal-ordered mesoporous matrix indexed as (100), (110), (200), and (210) Bragg peaks. Additionally, Figure 1b displays the PXRD of calcined MSN were a slight shift of the main (100) peak was observed, which corresponds to a cell contraction of ca. 3 Å due to the condensation of silanol groups in the calcination step. In a further step, Figure 1c shows the PXRD pattern of the final material **S1** obtained after loading the pores with linezolid and capping with PMB. A slight intensity reduction in the (100) reflection and the loss of the (110) and (200) reflections are observed, most likely due to the reduced contrast after the loading/functionalization process. Nevertheless, the permanence of the (100) reflection in the PXRD pattern strongly evidences that the mesoporous structure is maintained in the final gated nanoparticles as it can also be observed in TEM studies of calcined MSN (Figure 1d) and **S1** (Figure 1e). Representative TEM images of both solids showed spherical nanoparticles with a similar average diameter of ca. 110 nm. In addition, zeta potential measurements and dynamic light scattering (DLS) analysis were also used to characterize calcined MSN and **S1** nanoparticles. In this respect, the zeta potential shows values of −30.6 and 17.4 mV for calcined MCM-41 and **S1** nanoparticles, respectively. Additionally, hydrodynamic diameter values of 171 ± 4 and 252 ± 8 nm were found for the starting calcined MCM-41 and **S1** nanoparticles, respectively.

N_2_ adsorption–desorption isotherm studies were also performed. Specific surface area was calculated through the application of the Brunauer–Emmett–Teller (BET) model [39]. Pore size and pore volume was calculated using the Barret–Joyner–Halenda (BJH) model [40] on the adsorption branch of the isotherm. Calcined MSN showed the typical curve for mesoporous materials (Figure 1f), and a high surface area (1069 m^2^g^−1^) and pore volume (2.66 nm) were recorded (Table 1). In contrast, solid **S1** showed a reduced surface area (279 m^2^g^−1^) and no significant pore size due to the filling of mesopores and further external functionalization and capping processes (Figure 1g). Table 1 resumes the main structural characterization parameters for both materials.

Finally, the organic content of **S1** was determined through thermogravimetric and elemental analyses. Table 2 shows the organic content (mmol per gram of SiO_2_) of linezolid, carboxylate moieties, and PMB in the final material. With these data, linezolid and PMB encapsulation efficiency were calculated as 30% and 11%, respectively.

### 3.2. Controlled Release Studies

**Figure 2:**
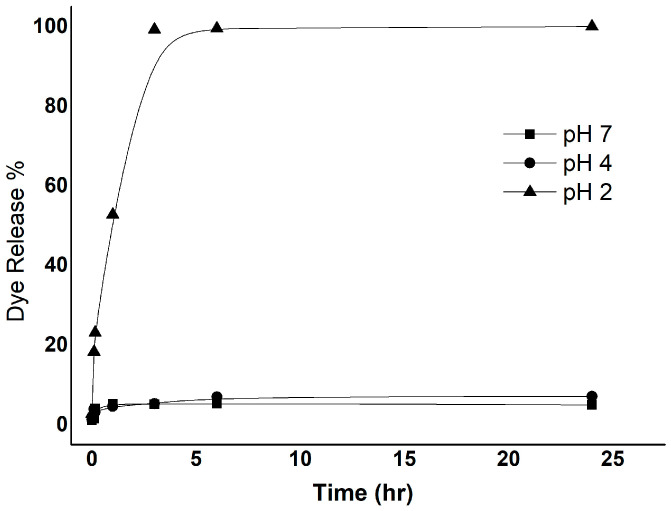
Kinetic release of RhB from **S1-Rh** at different pH levels.

In a further step, controlled-release experiments were undertaken to confirm that the electrostatic interaction between the anionic tricarboxylate derivative anchored in the solid surface and the capping cationic PMB was the force that prevented cargo release. Release mechanism analysis using pH changes was conducted using a rhodamine B (RhB)-loaded solid **S1-Rh** to mimic solid **S1**. A total of 1 mg of **S1-Rh** was suspended in 1 mL of distilled water solution at different pH levels (2, 4 and 7). At a certain time, aliquots were taken and centrifuged to eliminate the nanoparticles. The delivery of the rhodamine B dye was then monitored by measuring the fluorescence emission of RhB at 571 nm (λ_exc_ 555 nm). As shown in Figure 2, only a strong acidic pH was able to break these interactions, which resulted in rapid release of the dye.

**Figure 3:**
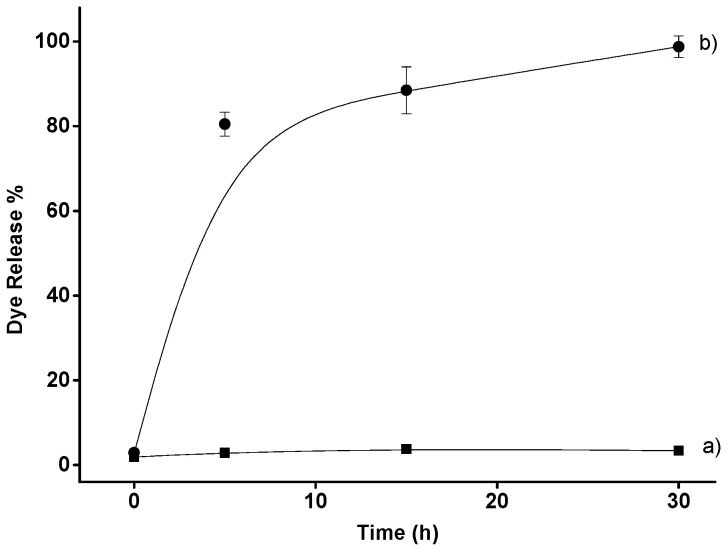
Kinetic release of RhB from **S1-Rh** (a) in the absence and (b) in the presence of LPS.

In our hypothesis, the recognition of PMB by LPS in the outer cell wall of bacteria was the stimulus that opened the gated nanoparticles. Likewise, the affinity of the PMB capping layer with LPS was tested using solid **S1-Rh**. A kinetic release using LPS was performed in aqueous solutions where 1 mg of **S1-Rh** was suspended in 1 mL of LPS-free phosphate buffer saline (PBS, pH 7.4) both in the presence of 2.5 mg of LPS and in its absence (control). At a certain time, the aliquots were separated and centrifuged. Delivery of the RhB dye to the bulk solution was then monitored through fluorescence (emission at 571 nm). As can be appreciated in Figure 3, no release of RhB was registered in the absence of LPS as a result of the strong interaction between the grafted tricarboxylate derivative and PMB. In contrast, the presence of LPS induced the fast release of the dye to the outer solution, which confirmed the gating mechanism associated with the LPS-PMB that triggers the displacement of PMB from the solid surface and allows cargo release.

**Figure 4:**
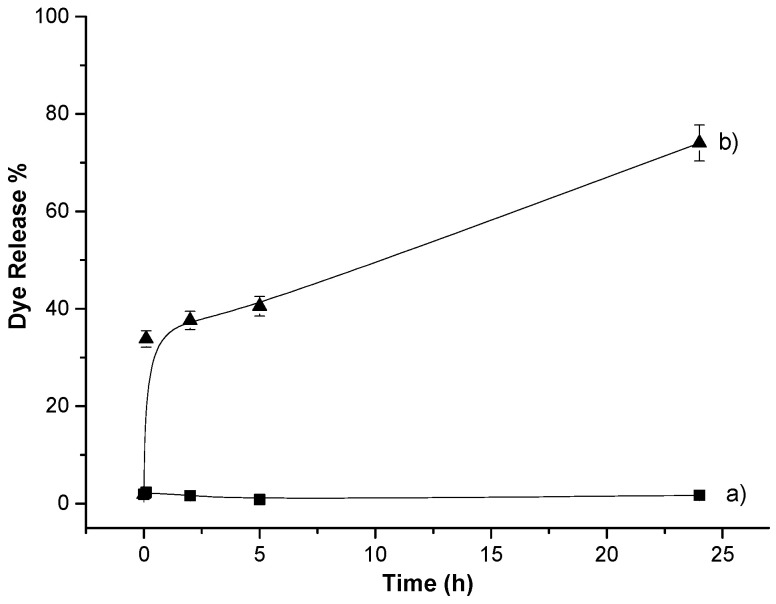
Kinetic release of RhB from **S1-Rh** (a) in the absence and (b) in the presence of bacteria.

Using the same experimental conditions, release profile in the presence of gram-negative bacteria *Escherichia coli* (DH5α strain) was tested. As can be observed in Figure 4, release of the encapsulated molecule from the gated material is inhibited in the absence of bacteria, but a cargo release was observed in the presence of bacteria, which confirmed the ability of the prepared nanoparticles to specifically release their content only in a bacterial environment. As described in the literature, PMB interacts with cell membrane LPS, thereby promoting its permeation [34]. After this step, linezolid can easily penetrate the bacteria and act with efficacy to kill the microorganism, as depicted in Figure 4. It is also expected that this selectivity of the prepared nanomaterials to target gram-negative bacteria will increase the concentration of the antibiotic in the cell surroundings and consequently increase the toxicity caused by linezolid.

While this is a first step, the development of this new nanoformulation still has a long way to go until it is applied in a clinical scenario. A comprehensive assessment of the interactions with cells, tissues, and organs should be addressed in further studies to investigate the appropriate dose and the identification of the best administration route to achieve the desired therapeutic effect [41].

### 3.3. Antimicrobial Efficacy Studies

**Figure 5:**
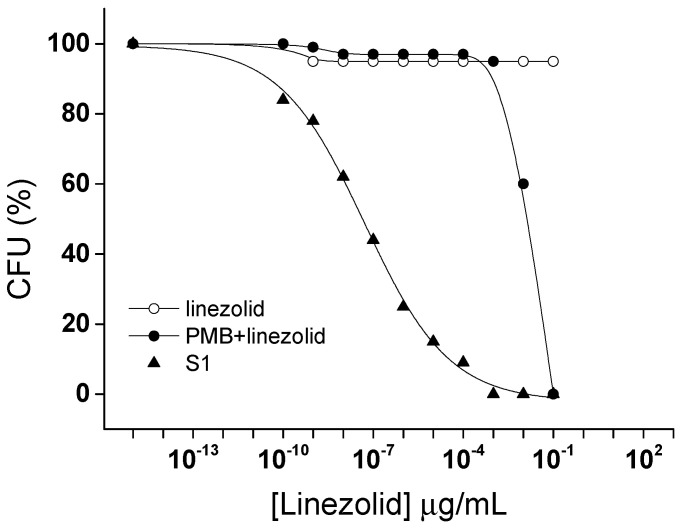
Clonogenic *E. coli* viability assay CFUS (%) treated at different concentrations of linezolid, the mixture of linezolid, and PMB and solid **S1**.

Once the gating mechanism was confirmed. Antimicrobial experiments with **S1** loaded with linezolid were performed. *E. coli* (DH5α strain) cell viability was tested upon increasing concentrations of **S1** (0 to 1 µg/mL in PBS at pH 7.4). Several controls, such as free linezolid, and a mixture of free linezolid with PMB at the equivalent concentrations as those in the nanoparticles were used. For viability studies, *E. coli* (DH5α strain) bacteria cells were enriched in LB agar and LB broth following the recommended conditions. After incubation overnight at 37 °C in LB medium under continuous stirring, bacteria from 1 mL of culture were collected through centrifugation and resuspended in 1 mL of PBS. Then, a dilution of 10^5^ cells/mL was prepared using turbidimetry measurements (OD_620_). Clonogenic viability assays of a series of dilutions of **S1**, linezolid, and the mixture of linezolid and PMB were performed. Each antimicrobial agent (**S1**, free linezolid or the mixture of linezolid and PMB) at the target concentration was mixed with the *E. coli* dilution and stirred for 10 min. Then, an aliquot of 100 µL was diluted at a ratio of 1:10 to obtain the final countable CFUS and seeded in a LB agar plate. All plates were incubated at 37 °C overnight. CFUS were counted in each plate, and the percentage of cytotoxicity was determined in comparison with a negative control of *E. coli* bacteria without antimicrobial treatment (100% viability). As can be appreciated in Figure 5, free linezolid was not toxic to the bacteria even at high concentrations of 1 µg/mL. This was expected as free linezolid is not toxic to gram-negative bacteria. When linezolid was mixed with equimolar concentrations of membrane disruptor PMB, a decrease in viability was found. A minimum inhibitory concentration (MIC) of around 1 × 10^−2^ µg/mL was estimated. It is noteworthy that when bacteria were treated with **S1** loaded with linezolid and capped with PMB, the calculated MIC was 10^5^ times lower (1 × 10^−7^ µg/mL) for **S1**.

These results confirmed that the **S1** nanomaterial works as a double-toxic agent. First, PMB interacts with LPS on the cell membrane, thereby causing an efficient membrane disruption [34] which facilitates the delivery of linezolid closer to the disrupted membrane and a more lethal result for the bacteria, as depicted in Figure 5. The nano-formulated combination of both antimicrobial agents in **S1** was found to be remarkably more efficient than the combination of both free linezolid and PMB. Moreover, mesoporous silica nanoparticles are recognized as very stable nanocarriers [42]. Nevertheless, their functionalization can affect their stability but not to a large extent. In this study, the prepared nanocarrier could be used even at 6 months after their preparation. The obtained results demonstrate the benefits of using this nano-delivery system to increase the toxicity of current antibiotics against pathogens.

**Figure 6:**
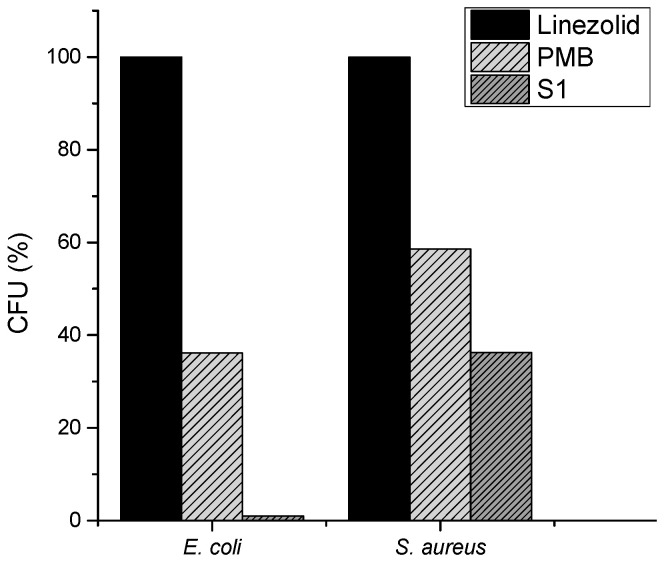
Bacterial viability (% CFU) of *E. coli* and *S. aureus* treated with 1 × 10^−4^ µg/mL of linezolid, PMB, and solid **S1**.

In a step forward, the feasibility of **S1** against gram-positive *S. aureus* compared with gram-negative *E. coli* was examined. A standard broth microdilution method in a 96-well plate reader was used to determine bacterial growth through OD_620_ and to calculate the antibacterial activity (MIC) (see Materials and Methods section for more details). For each bacterium, the viability percentage was determined in the presentence of **S1**, free PMB, and free linezolid at different concentrations; each sample was repeated three times. Linezolid alone was not toxic for *E. coli* (MIC > 1 µg/mL) and slightly toxic for *S. aureus* (MIC 1.22 × 10^−1^ µg/mL). Free PMB showed higher activity (MIC 1.95 × 10^−5^ µg/mL for *E. coli* and MIC 2.41 × 10^−4^ µg/mL for *S. aureus*). However, the **S1** nanomaterial caused 100-fold and 10-fold stronger growth inhibition than PMB in *E. coli* (MIC 1.23 × 10^−7^ µg/mL) and *S. aureus* (MIC 2.76 × 10^−5^ µg/mL), respectively. For example, as depicted in Figure 6, using only 1 × 10^−4^ µg/mL of **S1**, bacteria viability in both types of bacteria was remarkably reduced. From these experiments, the fractional inhibitory concentration index (FICI) can be calculated for each bacterium [43], which resulted in 0.0063 for *E. coli* and 0.12 for *S. aureus*, thus demonstrating the great synergy of the combination of linezolid and PMB in the developed nanoformulation. With this data, it can be concluded that the encapsulation of linezolid in a PMB-capped nanomaterial enhanced the antimicrobial efficiency against gram-positive and gram-negative bacteria.

### 3.4. Looking to the Future

In this work, we have described a novel nanoformulation to broaden linezolid effectivity and to have a new strategy for treating infections caused by bacteria. Using this nanoformulation, linezolid can permeate the infected cells and deliver higher concentration of antibiotic intracellularly. After this first attempt, the development of complex and validated models for intracellular infection together with a deeper understanding of the fate of bioinspired nanoantibiotics in infected cells will allow for the development of optimal nanoantibiotics that treat intracellular infection [44]. Deeper studies of how this nanoformulation can go through biological barriers, including changes in biodistribution, stability and protein adsorption, possible off-target toxicity due to the release of nanoparticles in other sites, or the pharmacokinetics and pharmacodynamics of the formulation should be studied in further research.

## 4. Conclusions

In summary, we report herein a new hybrid nanomaterial for the synergic delivery of antibiotics. Linezolid was loaded in silica mesoporous nanoparticles, and PMB was used to cap the pores of the nanomaterial and block linezolid release. When the nanomaterial is in the presence of bacteria, PMB interacts with high affinity with LPS on the bacteria surface and is displaced from the surface of the nanoparticles permeating the bacterium membrane and triggering the on-site release of linezolid. The antimicrobial activity of the prepared nano-delivery system was tested against gram-negative (*E. coli*) and gram-positive (*S. aureus*) bacteria. The results show that the prepared nanoformulation of **S1** enhanced the antimicrobial efficiency against these strains when compared with the unencapsulated linezolid and PMB. These results demonstrate the potential of using mesoporous silica nanomaterials as delivery systems for antibiotics with enhanced efficiency and targeted which ones might help to decrease the raising resistance against antibiotics, an important factor in overcoming antimicrobial resistance.

## Figures and Tables

**Table 1:** Main structural properties of nano-sized materials determined through TEM and N_2_ adsorption analysis.

Sample	Diameter Particle (nm)	Surface Area, *S*_BET_ (m^2^ g^−1^)	Pore Volume (cm^3^ g^−1^)	Pore Size (nm)
Calcined MSN	110	1011	0.86	2.66
**S1**	122	279	0.14	-

**Table 2:** Content (α) of encapsulated linezolid, anchored carboxylate moieties, and capping PMB in **S1**.

Solid	α_Linezolid_ (mmol g^−1^ SiO_2_)	α_Carboxylate moieties_ (mmol g^−1^ SiO_2_)	α_PMB_ (mmol g^−1^ SiO_2_)
S1	0.24	0.06	0.18

## Data Availability

Data will be made available on request.
